# TREC mediated oncogenesis in human immature T lymphoid malignancies preferentially involves *ZFP36L2*

**DOI:** 10.1186/s12943-023-01794-y

**Published:** 2023-07-10

**Authors:** Estelle Balducci, Thomas Steimlé, Charlotte Smith, Patrick Villarese, Mélanie Feroul, Dominique Payet-Bornet, Sophie Kaltenbach, Lucile Couronné, Ludovic Lhermitte, Aurore Touzart, Marie-Emilie Dourthe, Mathieu Simonin, André Baruchel, Hervé Dombret, Norbert Ifrah, Nicolas Boissel, Bertrand Nadel, Elizabeth Macintyre, Agata Cieslak, Vahid Asnafi

**Affiliations:** 1 Laboratory of Onco-Hematology, Necker Children’s Hospital, Assistance Publique-Hôpitaux de Paris (AP-HP), Paris, France; 2 Université Paris Cité, CNRS, INSERM U1151, Institut Necker Enfants Malades (INEM), Paris, France; 3 TAGC, UMR 1090, Aix-Marseille University, INSERM, Marseille, France; 4 Aix Marseille Université, CNRS, INSERM, CIML, Marseille, France; 5 Department of Pediatric Hematology and Immunology, University Hospital Robert Debré, Assistance Publique des Hôpitaux de Paris (APHP), Paris, France; 6 Institut Universitaire d’Hématologie, EA-3518, University Hospital Saint-Louis, Assistance Publique des Hôpitaux de Paris (APHP), Paris, France; 7 Université Paris Diderot, Institut Universitaire d’Hématologie, EA-3518, Assistance Publique-Hôpitaux de Paris, University Hospital Saint-Louis, 75010 Paris, France; 8 PRES LUNAM, CHU Angers Service Des Maladies du Sang, INSERM U 892, 49933 Angers, France

**Keywords:** T-cell receptor excision circles (TREC), Oncogenesis, Cancer

## Abstract

The reintegration of excised signal joints resulting from human V(D)J recombination was described as a potent source of genomic instability in human lymphoid cancers. However, such molecular events have not been recurrently reported in clinical patient lymphoma/leukemia samples. Using a specifically designed NGS-capture pipeline, we here demonstrated the reintegration of T-cell receptor excision circles (TRECs) in 20/1533 (1.3%) patients with T-cell acute lymphoblastic leukemia (T-ALL) and T-cell lymphoblastic lymphoma (T-LBL). Remarkably, the reintegration of TREC recurrently targeted the tumor suppressor gene, *ZFP36L2,* in 17/20 samples. Thus, our data identified a new and hardly detectable mechanism of gene deregulation in lymphoid cancers providing new insights in human oncogenesis.

**Supplementary Information:**

The online version contains supplementary material available at 10.1186/s12943-023-01794-y.

## Background

Adaptative immunity depends on V(D)J recombination to assemble antigen receptor genes from their component gene segments in T and B cells. During this process, the variable (V) diversity (D) and joining (J) gene segments present within the immunoglobulin (IG) and T-cell receptor (TCR) loci are assembled to form a complete V(D)J exon encoding the variable region of the IG/TCR. Recombination-activating gene (RAG) protein complexes consisting of heterotetrameric RAG1 and RAG2 proteins, recognize, capture and bind recombination signal sequences (RSSs) that flank V, (D) and J genes. Each RSS comprises a heptamer and a nonamer sequence separated by either 12 or 23 nucleotide spacer, which recombine according to the 12/23 rule. Following the capture of an RSS, the RAG protein complex generates a single strand break followed by 3'OH transesterification, forming a hairpin on coding ends (CE) and releasing a double strand DNA break (DSB) precisely at the junction between the RSS heptamer and the gene segment. Hairpin coding ends are nicked and processed to generate a coding joint by the non-homologous endjoining (NHEJ) pathway while blunt signal ends (SE) are fused into signal joints [1, 2]. Signal joints produced during V(D)J recombination are excised as episomal circles which are non-replicative but stable structures diluted through cell divisions [3].

Besides its essential role to provide a large antigen receptor repertoire in T and B cells, the V(D)J recombination machinery is also a threat to genomic stability, given its ability to induce DSB followed by erroneous repair of breaks in non-antigen receptor loci during the recombination process [4, 5]. Such aberrant recombination is involved in lymphoid oncogenesis, giving rise to translocations inducing activation of oncogenes as t(14;18)/*IGH-BCL2* in follicular lymphoma and t(11;14)/*IGH-CCND1* in mantle cell lymphoma or deletion of tumor suppressor genes such as *IKZF1* and *CDKN2A/B* in B and T-cell acute lymphoblastic leukemia (T-ALL) [6, 7].

In addition, it has been shown that in the presence of RAG, the excised episomal circles resulting from V(D)J recombination, previously considered inert, may be reintegrated into the genome through recombination occurring between the episomal signal joints and an IG/TCR target as well as into cryptic RSSs [3, 8–10]. The reintegration of such episomal circles in close proximity of oncogenes has been suggested as a mechanism of lymphoid oncogenesis by deregulation of target genes. However, this source of genomic instability has not been identified as a recurrent mechanism of oncogene deregulation in human lymphoid neoplasia. To investigate the reintegration of T-cell receptor excision circles (TREC) in clinical samples, we used a large T-ALL/T-cell lymphoblastic lymphoma (T-LBL) collection (*n* = 1533), to which we applied an NGS-capture pipeline designed to detect *TRD* (D and J gene segments) and partner gene translocations (Fig. S1). A specific bioinformatics pipeline was created to this aim as described schematically in the methods. For more details, the structural variant caller algorithm is available online https://github.com/Dr-TSteimle/sv-finder.

## Results and discussion

To evaluate the performance of our NGS pipeline, we took advantage of our previously published cohort of 264 T-ALL samples annotated for *TRD* translocations [4] which had been explored by fluorescence in situ hybridization (FISH) using a *TRD* dual-color probe. Concordant results were observed in 259/264 cases. NGS detected *TRD* translocations in 4 cases negative by FISH, all of which were confirmed by sequence specific PCR. Detailed sequence analysis revealed that two of them were not translocations but TREC insertion, as described below. The resulting sensitivity and specificity of the NGS pipeline compared to FISH were 98.1% [95% CI 96–99] and 97.7% respectively. The positive predictive value was 99.5% and the negative predictive value was 91.5%.

**Fig. 1:**
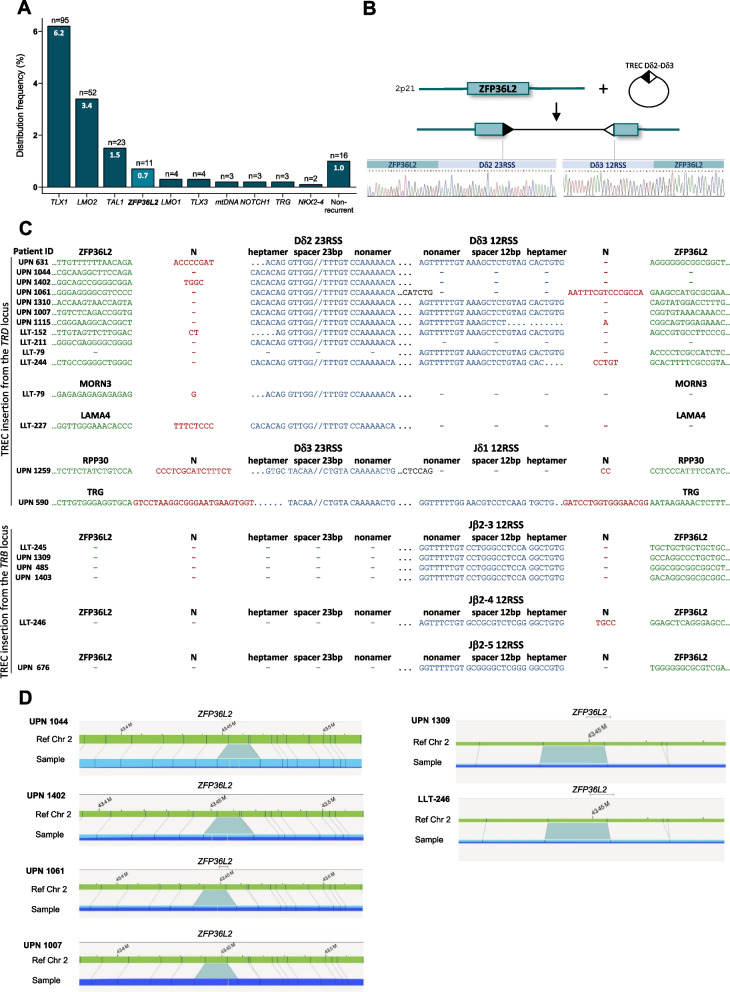
*TRD* translocations and TREC insertions from *TRD* and *TRB* loci in T-ALL/T-LBL patients. **A** Distribution frequency of the *TRD* partner genes among the 1533 T-ALL/T-LBL patients. *ZFP36L2*, the new recurrent partner gene, is indicated in light blue. **B** Schematic representation of insertion of TREC from *TRD* gene into the *ZFP36L2* gene. **C** Junction sequences of TREC insertion from *TRD* and *TRB* loci in *ZFP36L2*, *MORN3*, *LAMA4*, *RPP30* and *TRG* genes. **D** Fine mapping of chromosome 2 using optical genome mapping indicating an insertion sizing 10 Kb located in *ZFP36L2* on 2p21 in UPNs 1044, 1402, 1061, and 1007 and an insertion sizing 1.2 Kb located in *ZFP36L2* in UPNS 1309 and LLT-245

Next, we applied our NGS-capture pipeline to an additional discovery cohort of 1269 patients, leading to analysis of a total of 1533 T-ALL/T-LBL patients. Overall, 216 *TRD* translocations were detected in 209/1533 (13.6%) T-ALL/T-LBL patients (Fig. S1). While most patients (97%) exhibited one *TRD* translocation, a minority of cases (3%) had two *TRD* translocations involving two partner genes. No differences were observed in terms of incidence of *TRD* translocation comparing T-ALL and T-LBL. *TRD* partner genes were identified by NGS in all except one case, due to BLAST sequence failure. NGS identified the following recurrent *TRD* partner genes (i.e. observed in at least two patients): *TLX1* (*n* = 95, 44%), *LMO2* (*n* = 52, 24%), *TAL1* (*n* = 23, 11%), *ZFP36L2* (*n* = 11, 5%), *LMO1* (*n* = 4, 2%), *TLX3* (*n* = 4, 2%), mitochondrial DNA (mtDNA) (*n* = 3, 1%), *NOTCH1* (*n* = 3, 1%) and *NKX2-4* (*n* = 2, 1%) (Fig. 1A, Table S1). Additionally, trans-rearrangements involving *TRD* and *TRG* loci were observed in 3 cases, *TRB* locus in 1 case, and *IGH* locus in 1 case, all in T-ALL.

NGS enabled the identification of novel *TRD* partner genes such as *RPP30, ROCK1, CLX1/SNAI2, SORCS1, HOTAIR, MORN3, LAMA4,* and mitochondrial DNA (mtDNA)*.* All were confirmed using specific genomic PCR designed for each translocation (data not shown). From the newly identified *TRD*-translocated oncogenes, only the mtDNA was recurrent (in 3 cases of T-ALL). A recent publication reported the insertion of mtDNA into the nuclear genome with an occurrence of 1/10^3^ cancers mainly affecting various tumor genes such as *FHIT, CTNNA2, DDIT3, WIF1, BCL11B, KDM5A,* and *AKT2* [11]*.* Here, the inserted fragment from mtDNA originated from various areas of mtDNA. Schematic representations of *TRD* translocations and genomic position of breakpoints in partner gene are shown in Figs. S2, S3 and S4.

Among *TRD* partner genes, *ZFP36L2* was identified as a new recurrent partner gene, detected in a significant number of patients (*n* = 11, ~ 1% in T-ALL/T-LBL) (Fig. 1A, Table S1). To our knowledge only one publication using RNAseq reported *TRA/D-ZFP36L2* fusion transcripts in 2 cases of T-ALL [12]*.* Importantly, sequence analysis of the *TRD-ZFP36L2* breakpoint demonstrated that the genomic abnormalities detected by NGS in all 11 cases were in fact the reinsertion of TREC generated during Dδ2-Dδ3 rearrangements (Fig. 1B-C). TREC insertion sites in the *ZFP36L2* gene were not associated with cryptic RSS sequences suggesting that breaks in this gene were not RAG-induced. As first reported for chromosomal translocations, illegitimate V(D)J recombination has been reported to occur either through the targeting of a cryptic RSS (referred to as type 1 translocations), or through repair of V(D)J-initiated CE/SE with a chromosomal break devoid of such cryptic site (referred to as type 2 translocations) [13]. For type 2, although some non-RAG initiated breaks may occur in fragile sites (e.g. chromosome 18 breaks in t(14;18) translocations in follicular lymphoma [14]), the precise mechanisms producing the breaks are mostly unknown and may actually be quite diverse. Of note, we have previously reported than *TRB*- and *TRD*-oncogenes translocations in T-ALL are mostly of type 2 mechanism [4].

Regarding TREC reinsertion, both “type 1-like” and “type 2-like” mechanisms have also been recapitulated in vitro and in mouse models. In type 1-like reinsertions [3, 9, 10], all breaks are initiated by RAGs, and generate one signal joint and one pseudo-hybrid joint. In type 2-like reinsertions [10], breaks at the TCR loci are initiated by RAG variants (RAGcore) and breaks at the non-RSS locus are initiated enzymatically through the I-SceI endonuclease targeting an engineered I-SceI site. Broken ends are illegitimately repaired with SE, resulting in the generation of two pseudo-hybrid joints.

Mechanisms of type 1 and type 2 are theoretically similar for translocation and reintegration and experimental models predicted that both type 1-like and type 2-like TREC/B-cell receptor excision circles (BREC) reintegration should occur in humans. Indeed, our results validate the occurrence in humans of the “type 2-like” TREC reinsertions predicted by the engineered mouse models set up by Rommel et al. [10]. We further provide the proof of principle that such type 2-like TREC reinsertions, up to now observed as epiphenomena [10, 15], are actually recurrent events occurring at the vicinity of oncogenes, and potentially linked to their deregulation in T-ALL development.

From the 11 cases with *TRD* TREC insertion in *ZFP36L2* gene, for 6 cases, both RSS from Dδ2 and Dδ3 were identified using PCR-based sequencing. This suggests that the entire Dδ2-Dδ3 TREC has been reinserted. Of note, optical genome mapping (OGM) confirmed the presence of an insertion of ~ 10 kb in *ZFP36L2* at 2p21 (Fig. 1D) consistent with the entire Dδ2-Dδ3 TREC insertion in 4 cases (Table S3). Nevertheless, in several cases, the second RSS from either Dδ2 or Dδ3 has not been identified (Fig. 1C). This could be related to (i) PCR-based sequencing failure, (ii) junction sequence modifications induced by RAG/terminal deoxynucleotidyl transferase (TDT) activity including deletion/insertion, and (iii) we can not exclude that in some cases the TREC has been partially inserted. Further investigation, including Whole-Genome Sequencing or Oxford Nanopore long-read sequencing, would need to be performed to address these questions. Similar insertion in *ZFP36L2* was not detected using OGM on screening 141 hematological myeloid malignancies (acute myeloid leukemia *n* = 51, myelodysplastic neoplasm *n* = 44, chronic myelomonocytic leukemia *n* = 26, myeloproliferative neoplasm *n* = 20) or in NGS screening of 351 mature T-cell leukemia/lymphoma (acute T-cell leukemia/lymphoma *n* = 168, anaplastic large cell lymphoma *n* = 118, enteropathy associated T-cell lymphoma *n* = 25, other T-cell lymphoma *n* = 40). *ZFP36L2,* Zinc Finger Protein 36-like 2, which codes for an RNA-binding protein, is considered as a tumor suppressor gene in various cancers, including hematological malignancies [16]. In mice, lack of *ZFP36L1* and *ZFP36L2* in lymphocytes during thymopoiesis induces T-ALL transformation with dependence on the oncogenic transcription factor *NOTCH1* [17]. *ZFP36L2* has also been reported as a putative driver gene affected by mutations in T-ALL [18]. We further investigated the presence of TREC insertion from the *TRB* locus using an NGS pipeline designed to detect *TRB* rearrangements. Remarkably, we detected insertions of TREC from *TRB* in 6 patients in our cohort, all located in *ZFP36L2* (Fig. 1C, Table S2). In 2 cases with available material, OGM confirmed the presence of an insertion of ~ 1.2 kb located in *ZFP36L2* at 2p21 (Fig. 1D) consistent with the insertion of Dβ2-Jβ2–3 TREC (Table S3).

Patients with insertion of TREC from *TRD* and *TRB* loci were negative for known major oncogenes drivers (i.e. *TLX1*, *TLX3*, *HOXA9*, *TAL1*) in 11/13 patients, suggesting a potential role of TREC insertion on oncogenesis (Table S2). Moreover, immunophenotypic characterization demonstrated an early T-cell precursor (ETP) phenotype and/or immunogenotypic immaturity (IM) in 9/11 patients consistent with the fact that insertion of TREC into target genes occurs in an early progenitor.

Molecular mapping of other TCR breakpoints also revealed the insertion of TREC in 4 other partner genes: *MORN3*, *RPP30, LAMA4*, and *TRG* (Table S2) which have not been specifically explored within the context of the present manuscript but which demonstrate that TREC reintegration is not limited to the *ZFP36L2* gene.

## Conclusions

Using NGS-capture based analysis and a specific bioinformatics pipeline, our data demonstrated the proof of concept that D-J TREC reintegration is a recurrent and elusive mechanism of gene deregulation in T-ALL. This paves the way for further investigations, including the NGS panel designed to identify B-cell receptor excision circles (BREC) reinsertion in B-cell lymphoma/leukemia, and opens exciting novel perspectives in understanding of molecular mechanisms of human oncogenesis.

## Supplementary Information

**Additional file 1.** Supplementary materials.

**Additional file 2: Fig. S1.** Patient flow diagram. **Fig. S2.** Schematic representation of breakpoints in partner genes of *TRD *translocation. **Fig. S3.** Schematic representation of *TRD* translocations with *TLX1*, *LMO2* and *TAL1* oncogenes. **Fig. S4. **Schematic representation of *TRD* translocations with partner genes other than *TLX1*, *LMO2,* and *TAL1* (A) and trans-rearrangements involving *TRD* (B) excluding all TREC insertions which are shown in Fig. 1. **Table S1.** Incidence of recurrent and non-recurrent* TRD *translocation partner genes. **Table S2.** Clinical and biological characteristics of patients exhibiting insertion of TREC from *TRD* and *TRB* loci. **Table S3.** TREC size.

## Data Availability

All other data are available on reasonable request from the corresponding authors.

## Abbreviations

CE: Coding ends
BREC: B-cell receptor excision circles
DSB: Double strand DNA break
ETP: Early T-cell precursor
FISH: Fluorescence in situ hybridization
IG: Immunoglobulin
IM: Immunogenotypic immaturity
mtDNA: Mitochondrial DNA
NGS: Next-generation sequencing
NHEJ: Non-homologous end joining
OGM: Optical genome mapping
RAG: Recombination-activating gene
RSS: Recombination signal sequences
SE: Signal ends
T-ALL: T-cell acute lymphoblastic leukemia
TCR: T-cell receptor
TDT: Terminal deoxynucleotidyl transferase
T-LBL: T-cell lymphoblastic lymphoma
TREC: T-cell receptor excision circles
