# Supplementary material for: TREC mediated oncogenesis in human immature T lymphoid malignancies preferentially involves ZFP36L2
Source: Mol Cancer. 2023 Jul 10;22:108. doi: 10.1186/s12943-023-01794-y (PMC10332067; doi:10.1186/s12943-023-01794-y)
Supplement: Supplementary file 1 — Additional file 1. Supplementary materials. [file 12943_2023_1794_MOESM1_ESM.docx]

# **Supplementary materials**

**Patient samples**

Diagnostic peripheral blood or bone marrow samples from a cohort of 1533 patients (1177 T-ALL and 356 T-LBL) were collected after informed consent was obtained according to the Declaration of Helsinki with approval from the institutional review boards of institutions that participated to the study.

**NGS**

Nextera XT (Illumina) DNA Libraries were prepared according to the manufacturer’s instructions, and sequenced using the Illumina NextSeq sequencing system, as previously described[1]. Sequencing reads were analysed using an in-house bioinformatics pipeline designed to detect structural variants (SVs).

**Structural variants caller algorithm**

To detect and categorize SVs from paired-end short reads, we developed a software called Sv-finder. For more in-depth comprehension, the open-sourced code is available online at <https://github.com/Dr-TSteimle/sv-finder>. Sequenced reads were aligned to the reference genome with bwa mem default parameters. The resulting bam file was used as input. The steps of the algorithm of Sv-finder are listed below. 1) First the software reads the bam file and keeps abnormal reads which are mapped with the wrong insert size (sam flags: 81, 161, 97, 145, 65, 129, 113, 177) or with supplementary alignment in auxiliary data (<https://samtools.github.io/hts-specs/SAMv1.pdf>). 2) These filtered reads were then clustered together owing to their distance on the reference genome for all their positions (primary and supplementary alignments). The minimum distance chosen for segregating reads was set to 350 nt. Thus, each cluster contains reads having the same misalignment patterns. 3) Overlapping R1 and R2 were assembled if they share at least 20 nt at one of their ends. 4) Inside each cluster the algorithm draws an undirected graph, linking together reads if they share an overlapping sequence of at least 20 nt with a tolerance of 5 non-consecutive mismatches and only one consecutive mismatch. 5) The generated graph was then traversed and the sequence without any neighbour overlapping its left side was selected as the start of the consensus sequence. From this stem sequence, each of its right overlapping neighbours was stacked, and neighbour by neighbour, the stack was stretched from left to right. Once all the sequences in the graph had been stacked, a consensus sequence was established in each column of the stack, when the absolute majority of read bases were identical and covered by at least two reads. 6) The consensus sequence was then aligned to the reference genome (bwa mem). Analysis of the alignment results gave a breakpoint position. Finally, a Human Genome Variation Society (HGVS) unique identifier for the SV was coined (<https://varnomen.hgvs.org/recommendations/general/>).

**FISH analysis**

FISH analysis for *TRD* rearrangement from our previously published cohort of 264 T-ALL samples was performed using a dual-color probe designed with the following BAC clones: CTD-2552B11 and RP11-1083M21 for the *TRD* gene.

**Optical genome mapping**

Optical genome mapping was performed as previously described[2]. Briefly, for each sample, 1.5 million cells from BM aspirates or PB samples were used to purify ultra-high molecular weight DNA using the Bionano Prep SP BMA DNA Isolation kit (Bionano Genomics). Then, DNA extraction, labeling, chip loading and data collection were performed following manufacturer instructions (Bionano Genomics). All data were analyzed using the rare variant pipeline from Bionano Genomics. The rare variant pipeline was executed on Bionano Solve software V3.5. Direct visualization and interpretation of SVsand copy number variations weredone on Bionano Access V1.7.

**Immunophenotypic and molecular characterization**

Analyses for immunophenotype, SIL-TAL fusion transcripts, HOXA9 overexpression, oncogenic transcripts (*TLX1, TLX3*), T-cell receptor (TCR) recombination, and *NOTCH1/FBXW7/RAS/PTEN* mutations were performed as previously described [3–5]. Patients were classified as immature (negative for TCR and cTCRβ), TCRαβ and TCRγδ.

**Statistical analysis**

Chi^2^ analysis was used as appropriate to make comparisons. Statistical analysis was performed with GraphPrism V8. p-values were two-sided with p<0.05 considered statistically significant.

**Supplementary references**

1. Bond J, Graux C, Lhermitte L, Lara D, Cluzeau T, Leguay T, et al. Early Response-Based Therapy Stratification Improves Survival in Adult Early Thymic Precursor Acute Lymphoblastic Leukemia: A Group for Research on Adult Acute Lymphoblastic Leukemia Study. J Clin Oncol Off J Am Soc Clin Oncol. 2017;35:2683–91.

2. Balducci E, Kaltenbach S, Villarese P, Duroyon E, Zalmai L, Friedrich C, et al. Optical genome mapping refines cytogenetic diagnostics, prognostic stratification and provides new molecular insights in adult MDS/AML patients. Blood Cancer J. 2022;12:126.

3. Asnafi V, Beldjord K, Boulanger E, Comba B, Le Tutour P, Estienne M-H, et al. Analysis of TCR, pT alpha, and RAG-1 in T-acute lymphoblastic leukemias improves understanding of early human T-lymphoid lineage commitment. Blood. 2003;101:2693–703.

4. Bergeron J, Clappier E, Radford I, Buzyn A, Millien C, Soler G, et al. Prognostic and oncogenic relevance of TLX1/HOX11 expression level in T-ALLs. Blood. 2007;110:2324–30.

5. Trinquand A, Tanguy-Schmidt A, Ben Abdelali R, Lambert J, Beldjord K, Lengliné E, et al. Toward a NOTCH1/FBXW7/RAS/PTEN-based oncogenetic risk classification of adult T-cell acute lymphoblastic leukemia: a Group for Research in Adult Acute Lymphoblastic Leukemia study. J Clin Oncol Off J Am Soc Clin Oncol. 2013;31:4333–42.
