# Supplementary material for: TREC mediated oncogenesis in human immature T lymphoid malignancies preferentially involves ZFP36L2
Source: Mol Cancer. 2023 Jul 10;22:108. doi: 10.1186/s12943-023-01794-y (PMC10332067; doi:10.1186/s12943-023-01794-y)
Supplement: Supplementary file 2 — Additional file 2: Fig. S1. Patient flow diagram. Fig. S2. Schematic representation of breakpoints in partner genes of TRD translocation. Fig. S3. Schematic representation of TRD translocations with TLX1, LMO2 and TAL1 oncogenes. Fig. S4. Schematic representation of TRD translocations with partner genes other than TLX1, LMO2, and TAL1 (A) and trans-rearrangements involving TRD (B) excluding all TREC insertions which are shown in Fig. 1. Table S1. Incidence of recurrent and non-recurrent TRD translocation partner genes. Table S2. Clinical and biological characteristics of patients exhibiting insertion of TREC from TRD and TRB loci. Table S3. TREC size. [file 12943_2023_1794_MOESM2_ESM.docx]

**Supplementary information**


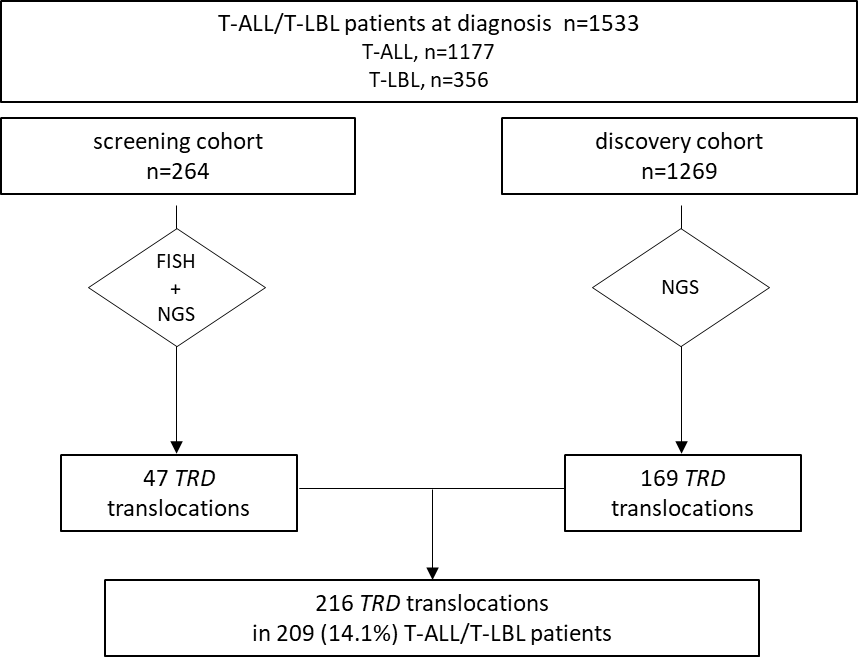


**Fig. S1 Patient flow diagram.**

###
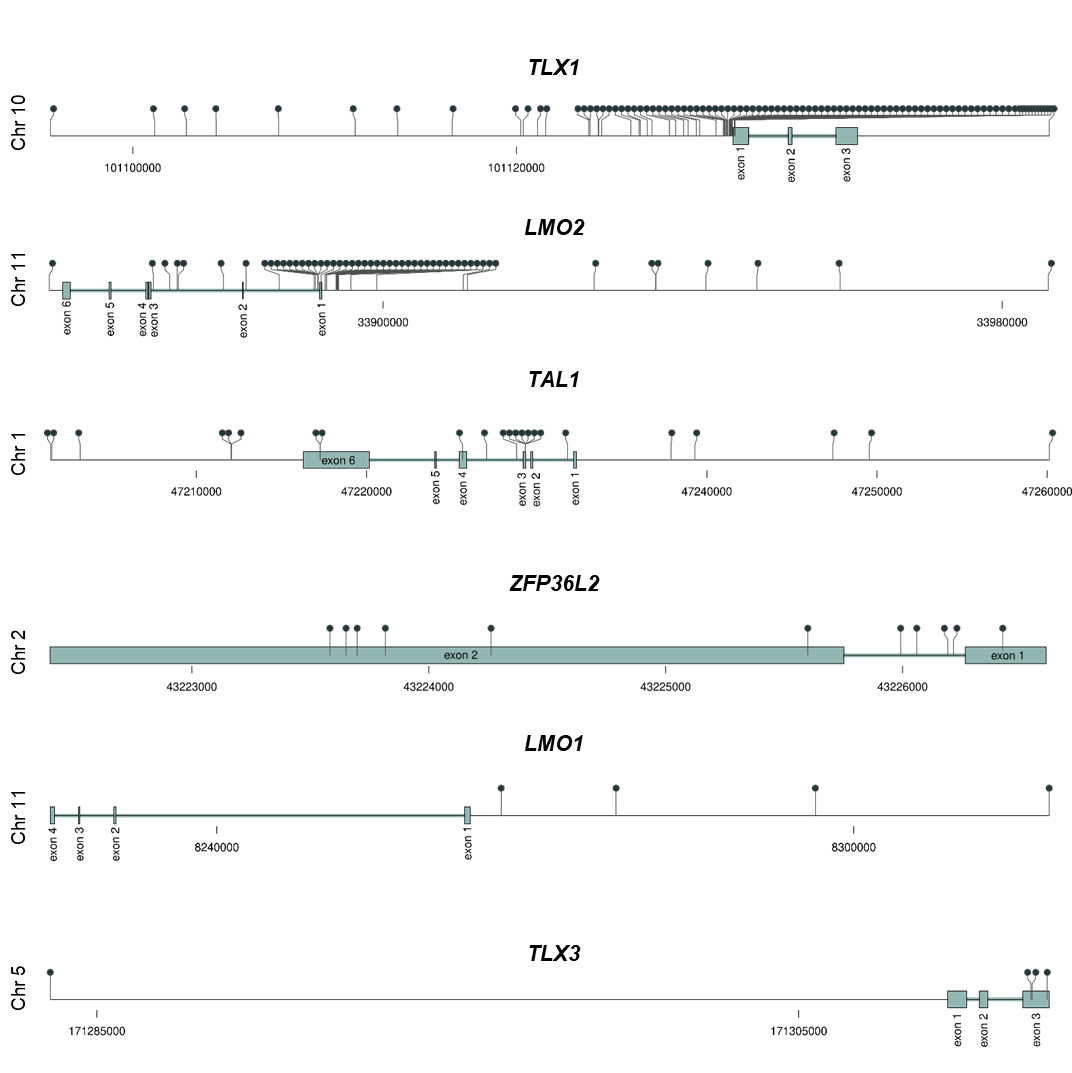


### **Fig. S2** **Schematic representation of breakpoints in partner genes of *TRD* translocation.**

Lollipop markers indicate the relative positions of breakpoints within *TLX1*, *LMO2*, *TAL1*, *ZFP36L2*, *LMO1* and *TLX3* genes.


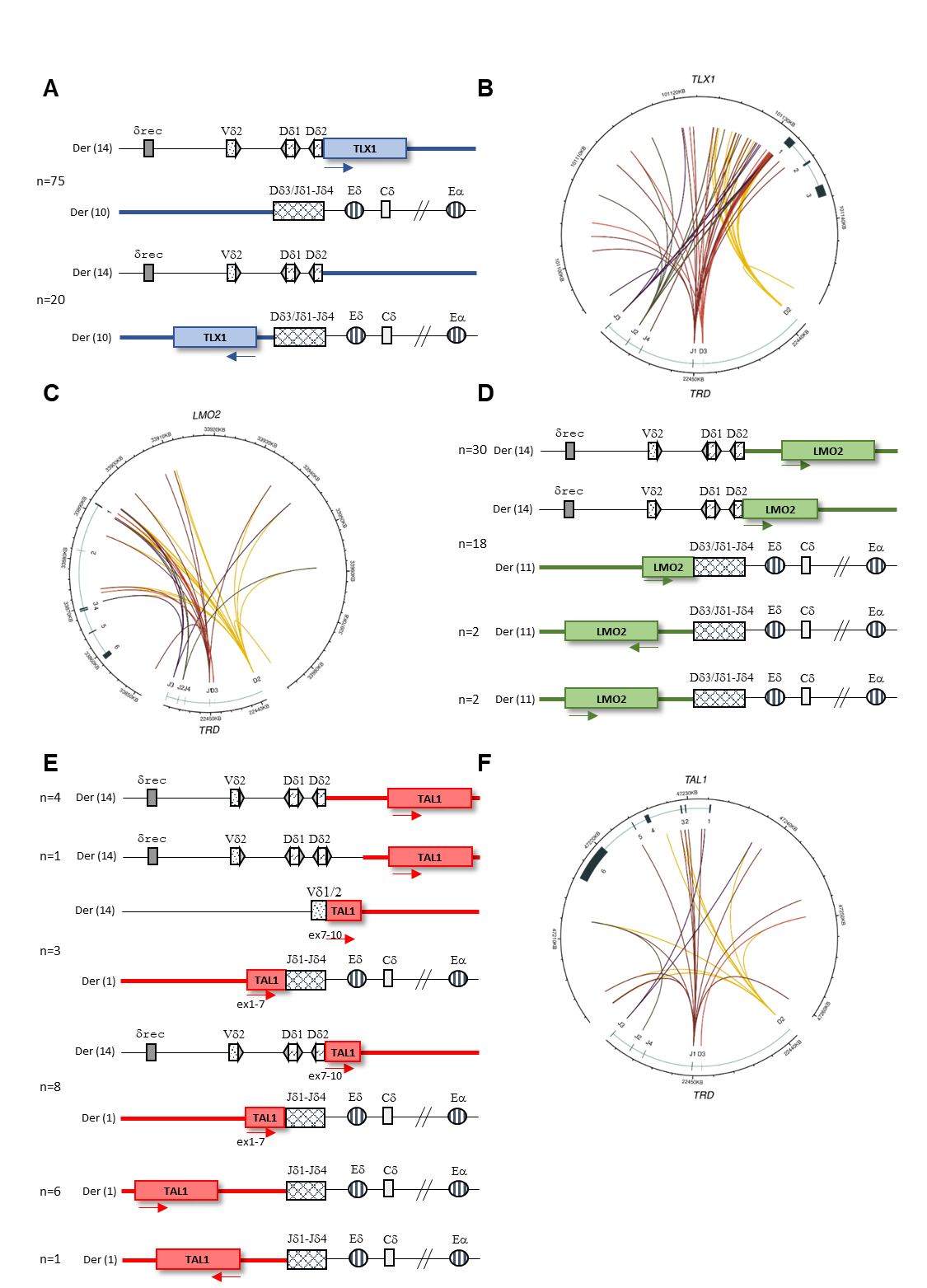


### **Fig. S3** **Schematic representation of *TRD* translocations with *TLX1*, *LMO2* and *TAL1* oncogenes.**

*TRD* translocation with *TLX1* (A-B), *LMO2* (C-D), and *TAL1* (E-F) partner genes. Thin and bold bars depict the *TRD* locus and partner gene locus, respectively. Circos plots show the locations of breakpoints in *TRD* locus and partner genes.


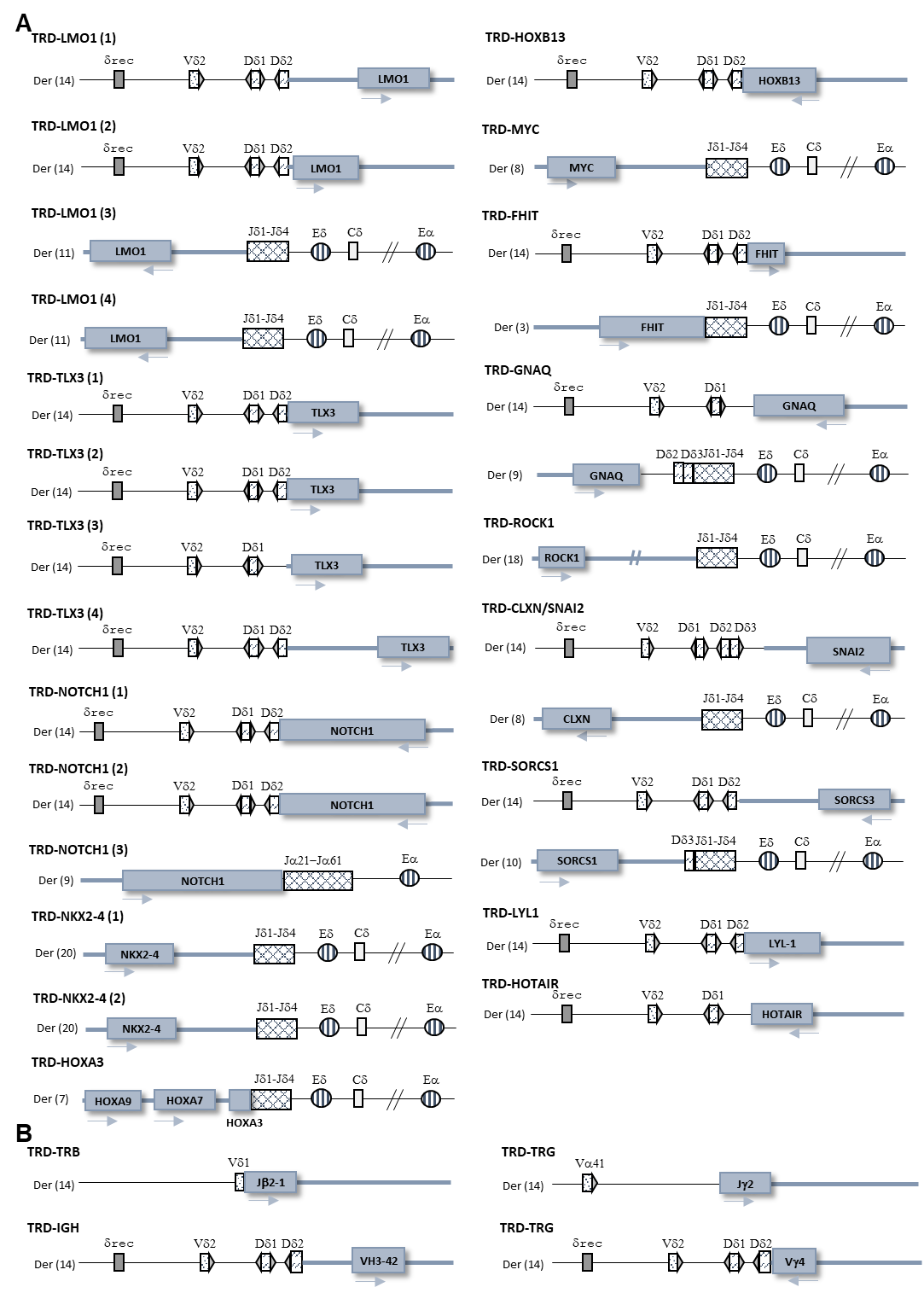


**Fig. S4**

Schematic representation of *TRD* translocations with partner genes other than *TLX1*, *LMO2,* and *TAL1* (A) and trans-rearrangements involving *TRD* (B) excluding all TREC insertions which are shown in Fig.1. Thin and bold bars depict the *TRD* locus and partner gene locus, respectively. Only derivatives with partner genes are shown.


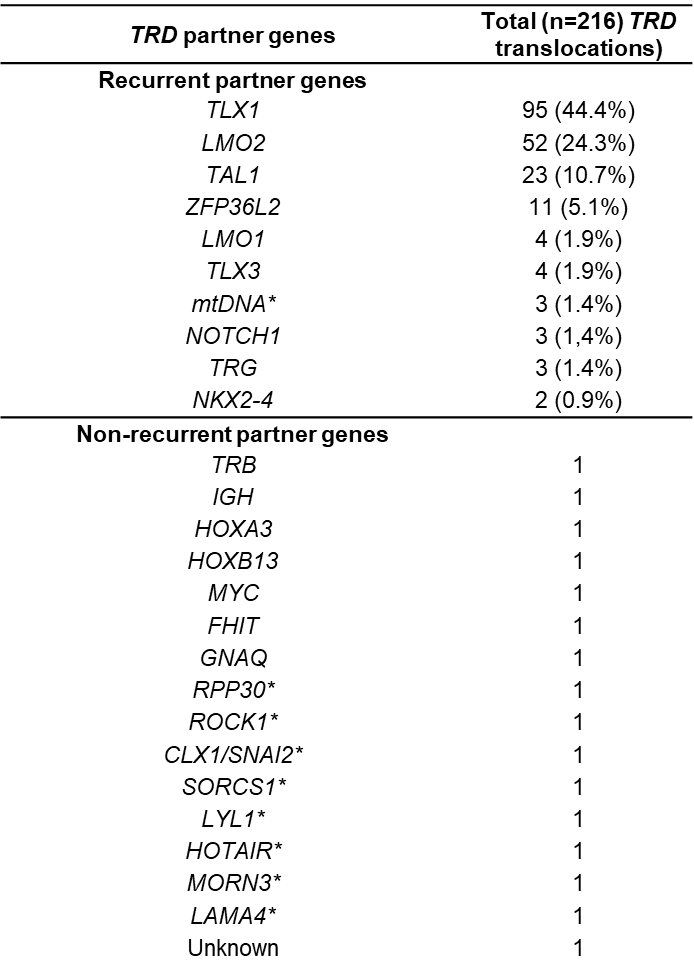


**Table S1 Incidence of recurrent and non-recurrent *TRD* translocation partner genes.**

Percentages were calculated amongst cases with *TRD* translocations. Asterisks indicate newly identified partner genes.


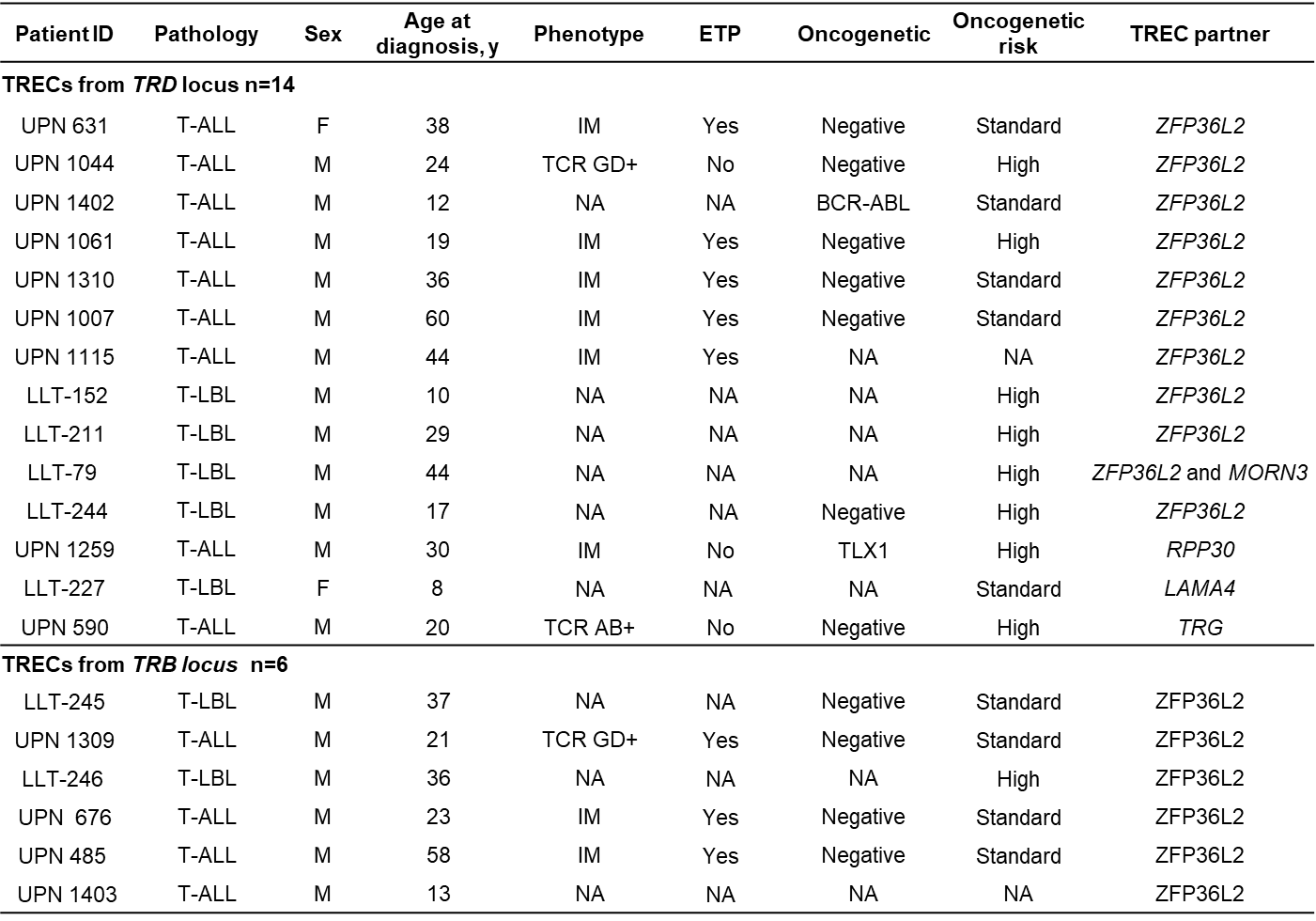


**Table S2 Clinical and biological characteristics of patients exhibiting insertion of TREC from *TRD* and *TRB* loci.**

Negative indicates cases with neither PICALM-MLLT10, STIL-TAL1 fusion transcript nor *HOXA9/TLX1/TLX3* overexpression. y, years; NA, not available; IM, immature phenotype with absence of TCR and cTCRβ.

| **TREC** | **Size** |
| --- | --- |
| Dδ2-Dδ3 | 10098 bp |
| Dδ3-Jδ1 | 963 bp |
| Dβ2-Jβ2-3 | 1127 bp |
| Dβ2-Jβ2-4 | 1278 bp |
| Dβ2-Jβ2-5 | 1399 bp |
| Dβ1-Jβ2-3 | 10623 bp |
| Dβ1-Jβ2-4 | 10774 bp |
| Dβ1-Jβ2-5 | 10898 bp |

**Table S3 TREC size.**

The size of the insert if the TREC was inserted intact. For Jβ2 gene segments, the theoretical TREC size is indicated if the segment is rearranged directly with Dβ2 or Dβ1 gene segment.
